# Association between gestational blood lipids and TSH levels and pregnancy outcome of patients with subclinical hypothyroidism

**DOI:** 10.12669/pjms.39.3.7150

**Published:** 2023

**Authors:** Jiajia Zhang, Hao Chen, Xiaobing Dou, Wei Huang, Haixia Zeng

**Affiliations:** 1 Jiajia Zhang Department of Obstetrics, The First Affiliated Hospital of Wenzhou Medical University, Wenzhou 325000, Zhejiang Province, P.R. China; 2 Hao Chen Department of Thyroid Surgery, The First Affiliated Hospital of Wenzhou Medical University, Wenzhou 325000, Zhejiang Province, P.R. China; 3 Xiaobing Dou Department of Pediatric Internal Medicine, The First Affiliated Hospital of Wenzhou Medical University, Wenzhou 325000, Zhejiang Province, P.R. China; 4 Wei Huang Department of Obstetrics, The First Affiliated Hospital of Wenzhou Medical University, Wenzhou 325000, Zhejiang Province, P.R. China; 5 Haixia Zeng Department of Obstetrics, The First Affiliated Hospital of Wenzhou Medical University, Wenzhou 325000, Zhejiang Province, P.R. China

**Keywords:** Subclinical hypothyroidism, Pregnancy, Blood lipids, Thyroid stimulating hormone, Pregnancy outcome

## Abstract

**Objective:**

To investigate the association between gestational blood lipids and thyroid stimulating hormone (TSH) levels and pregnancy outcomes of patients with subclinical hypothyroidism (SCH).

**Methods:**

In this retrospective observational study, we analyzed the clinical data of 82 patients (case group) with gestational SCH treated in our hospital from January 2021 to January 2022 at gestational weeks 25-33 and grouped them according to whether SCH was well controlled by treatment (case Group-A: well controlled, n=55; case Group-B: poorly controlled, n=27), and the clinical data of 41 pregnant women (control group) undergoing physical examination during the same period. After comparing the blood lipids and TSH levels of the three groups, we compared their adverse pregnancy outcomes to assess the possible correlations between blood lipids and TSH levels and pregnancy outcomes.

**Results:**

The levels of total cholesterol (TC), triglyceride (TG), low density lipoprotein cholesterol (LDL-C) and TSH in the case Group-B were significantly higher than those in the case Group-A and the control group (P<0.05). Compared with case Group-B and the control group, the incidence of premature delivery, abortion and neonatal growth restriction was higher in case Group-A (*P*<0.05). Among 82 patients in the case group 42 presented adverse pregnancy outcomes. The levels of TC, TG, LDL-C and TSH in mothers and infants in the adverse outcome group were significantly higher than those in the favorable outcome group (*P*<0.05). Our Pearson analysis results showed that the levels of TC, TG and LDL-C were positively correlated with the TSH levels and the pregnancy outcomes, and that TSH was positively correlated with pregnancy outcomes (*P*<0.05).

**Conclusion:**

The levels of TC, TG, LDL-C and TSH in patients with poorly controlled SCH were increased during pregnancy, and were associated with the pregnancy outcomes and positively correlated with each other.

## INTRODUCTION

Subclinical hypothyroidism (SCH) is a common metabolic disease in pregnant women with TSH levels above 4.5-5.0mlu/l and free thyroid hormone levels within normal ranges.^1^ The incidence of SCH is approximately 10%, and most patients do not have symptoms or signs of the disease. The main cause of SCH is usually assumed to be related to an autoimmune dysfunction directed at the thyroid and is often ignored.^2^,^3^ Some scholars have speculated that the morbidity and mortality of patients with SCH are associated with the effects of the condition on blood lipid levels, but it is still controversial.^4^,^5^ In addition, clinical treatment guidelines for patients with abnormal blood lipids levels and SCH are lacking.^6^

SCH during pregnancy presents with an abnormal thyroid function, which can affect blood marker levels, and metabolism, circulatory and other systems factors. Moreover, normal thyroid hormone levels are important for the fetal nervous tissues’ growth and development, being important to maintain a normal metabolism and to regulate lipid metabolism.^7^ The blood lipids levels of pregnant women have a direct impact on the growth and development of the fetus. High blood lipids levels can induce fetal ischemia and hypoxia, resulting in fetal respiratory distress, Fmalformations and growth restriction, factors that negatively affect the outcome of a fetus.^8^ Several studies have indicated that SCH during pregnancy can affect fetal neurodevelopment and that it increases the risk of adverse pregnancy outcomes.^9^,^10^ However, few reports on the gestational blood lipids levels of women with SCH exist, and the association between gestational blood lipids and TSH levels in these patients and pregnancy outcomes remain unclear. Therefore, the purpose of this study was to explore the associations between the gestational levels of blood lipids and TSH in patients with SCH and their pregnancy outcomes, so as to provide a basis for clinicians to treat patients with SCH during pregnancy.

## METHODS

In this retrospective observational study, we retrospectively selected data from 82 women with gestational SCH treated in our hospital from January 2021 to January 2022 at gestational weeks 25-33 and they were grouped according to whether SCH was well controlled by treatment (case Group-A: well controlled, n=55; case Group-B: poorly controlled, n=27, and clinical data from 41 healthy pregnant women from the same period as the control group. The medical ethics committee of our hospital approved this study (Approval number YS2022-224; May 5, 2022)

### Inclusion criteria:

- We applied the diagnostic criteria of the 2017 American Thyroid Association guidelines for the diagnosis and treatment of thyroid diseases during pregnancy, with serum TSH levels between 2.5 and 10 mU/L and serum T4 concentrations between 12 and 22 pmol/L.^11^
- Single pregnancies were diagnosed at gestational weeks 25-33 on the basis of color ultrasound images.
- All participants had complete clinical data.

### Exclusion criteria:

Patients complicated with pregnancy complications, metabolic diseases, liver or kidney dysfunction, preeclampsia, gestational diabetes and hypertension.

- Patients with history of autoimmune, infectious diseases, thyroid diseases and thyroidectomy.
- Patients with coagulation dysfunction or malignant tumor.

### Observation index:

We collected background clinical data from the patients in the case and the control groups (age, gestational weeks, body mass index, blood pressure levels, and others). Blood lipids and TSH levels (from 5-ml samples of overnight-fasting venous blood analyzed in a Boke automatic biochemical analyzer bk-600). Blood markers and their normal ranges included TC (2.9-6.1 mmol/L), TG (0.56-1.7 mmol/L), HDL-C (1.16-1.55 mmol/L), LDL-C (2.07-3.37 mmol/L) and TSH (0.24-4.2 mU/L). *Treatment protocol:* Immediately after the diagnosis of SCH, treatment should be carried out, including life and diet guidance for pregnant women, maintaining a balanced diet, strengthening iodine intake, maintaining adequate sleep time and quality, and then giving levothyroxine sodium tablets (National Medicine Approval H20010008, Shenzhen Zhonglian Pharmaceutical Co., Ltd., specification: 25μg) 25~50μg orally on an empty stomach. Thyroid function was checked every two weeks. Doctors adjusted the drug dose according to the TSH level, and the patients kept regular medication during pregnancy until delivery. TSH level control within 4.0mU/L was considered well control, and vice versa is considered poor control. We recorded adverse pregnancy outcomes of the women (postpartum hemorrhage, premature rupture of membranes, premature delivery and abortion) and adverse pregnancy outcomes of their newborns (respiratory distress, growth restriction and malformations).

### Statistical analysis:

The sample size was estimated based on a prevalence of SCH during pregnancy between 1.5% to 42.9%, with a 2-sided significance level of .05 and a 10% possibility of incomplete clinical records, at least 26 patients were required.^12^,^13^ We used the SPSS 24.0 software for our analyses. The measurement data conforming to the normal distribution are represented by means and standard deviations (*χ̅*±*S*), and we applied t-tests for comparisons, Wilcoxon rank sum test was used for data with non-normal distribution; the counting data is represented by numbers and percentages [n (%)], and we applied *χ^2^* inspection for comparisons. We assessed the association between levels of blood lipids and TSH and the pregnancy outcomes using Pearson correlation analyses.

## RESULTS

There were no significant differences in basic data variables between the groups (*P*>0.05), Table-I. We found similar HDL-C levels between the two groups (*P*>0.05). The levels of TC, TG, LDL-C and TSH in the case Group-B were significantly higher than those in the case Group-A and the control group (*P*<0.05), Table-II.

**Table-I:** Comparison of basic data between the groups (*χ̅*±*S*).

Groups	n	Age (years)	Gestational week	BMI (kg/m^2^)	Blood pressure (mmHg)	

					Systolic pressure	Diastolic pressure
Case Group-A	55	29.41±4.91	28.48±3.48	25.24±1.86	117.19±11.42	71.40±5.84
Case Group-B	27	29.23±4.76	28.65±3.43	25.28±1.74	118.35±11.23	71.34±5.86
Control group	41	28.95±4.83	29.04±3.72	25.02±2.19	119.34±12.58	69.41±6.84
F		0.111	0.237	0.199	0.397	1.383
p		0.895	0.790	0.820	0.674	0.255

**Table-II:** Comparison of blood lipids and TSH levels between the groups (*χ̅*±*S*).

Groups	n	Blood lipid				TSH(mU/L)

		TC(mmol/L)	TG(mmol/L)	HDL-C(mmol/L)	LDL-C(mmol/L)	
Case Group-A	55	5.54±0.70	1.53±0.33	1.53±0.14	3.42±0.51	2.12±0.38
Case Group-B	27	5.98±0.75^#^	1.76±0.56^#^	1.54±0.12	3.86±0.62^#^	2.85±0.48^#^
Control group	41	5.31±0.52	1.42±0.38	1.52±0.16	3.23±0.50	1.95±0.52
F		26.930	5.757	0.162	11.583	34.759
p		<0.001	0.004	0.851	<0.001	<0.001

# p<0.05 compared with the control group.

**Fig.1:**
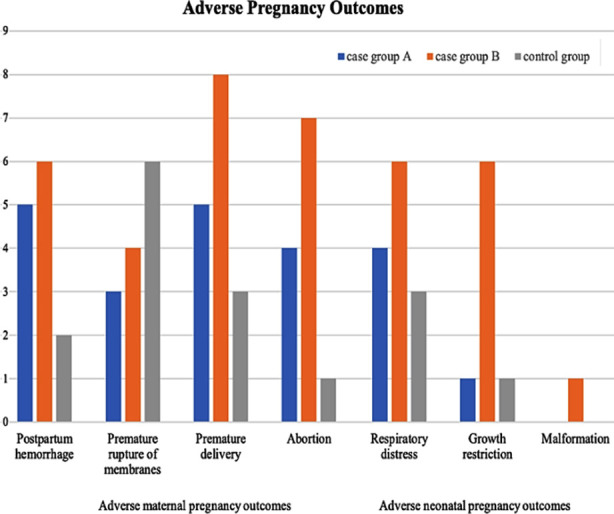
Histogram of adverse pregnancy outcomes in the three groups.

There was no statistically significant difference in the incidence of postpartum hemorrhage, premature rupture of membranes, neonatal respiratory distress and malformation between the groups (P > 0.05). Compared with case Group-B and the control group, the incidence of premature delivery, abortion and neonatal growth restriction was higher in case Group-A (*P*<0.05), Table-III. Among 82 patients, 42 presented adverse maternal and neonatal outcomes and 40 had favorable outcomes. The levels of TC, TG, LDL-C and TSH of the women with adverse pregnancy outcomes were higher than those of the women with favorable outcomes (*P*<0.05), Table-IV.

**Table-III:** Comparison of adverse pregnancy outcomes between the groups [n (%)].

Groups	n	Adverse maternal pregnancy outcomes				Adverse neonatal pregnancy outcomes		

		Postpartum hemorrhage	Premature rupture of membranes	Premature delivery	Abortion	Respiratory distress	Growth restriction	Malformation
Case Group-A	55	5(9.09)	3(5.45)	5(9.09)*	4(7.27)*	4(7.27)	1(1.82)*	0
Case Group-B	27	6(22.22)	4(14.81)	8(29.63)^#^	7(25.93)^#^	6(22.22)	6(22.22)^#^	1(3.70)
Control group	41	2(4.88)	6(14.63)	3(7.32)	1(2.44)	3(7.32)	1(2.44)	0
*χ* ^2^		5.411	2.754	8.511	10.987	4.970	14.070	3.585
p		0.067	0.252	0.014	0.004	0.083	0.001	0.167

* p < 0.05 compared with case Group-B;

# p < 0.05 compared with the control group.

**Table-IV:** Comparison of levels of blood lipids and TSH levels in the case groups according to different pregnancy outcomes (*χ̅*±*S*).

Pregnancy outcome	n	Blood lipids			TSH (mU/L)

		TC(mmol/L)	TG(mmol/L)	LDL-C(mmol/L)	
Adverse outcomes	42	6.33±0.66	1.78±0.34	3.63±0.51	6.35±1.08
Favorable outcomes	40	5.61±0.63	1.59±0.23	3.29±0.46	5.61±0.90
*t*		5.048	2.949	3.165	3.362
p		<0.001	0.004	0.002	0.001

According to our Pearson analysis results, the levels of TC, TG and LDL-C were positively correlated with the TSH level and the pregnancy outcomes, and the level of TSH was positively correlated with the pregnancy outcomes (*P*<0.05), Table-V.

**Table-V:** Correlation Analysis between levels of blood lipids and TSH and pregnancy outcomes

Index	TSH		Pregnancy outcome	

	r	p	r	p
TC	0.554	<0s.001	0.365	0.001
TG	0.359	<0.001	0.218	0.049
LDL-C	0.233	0.009	0.242	0.029
TSH	-	-	0.240	0.030

## DISCUSSION

High levels of blood lipids have been associated with the presence of SCH during pregnancy. In this study, we collected and analyzed the blood lipids and TSH levels of pregnant women with or without gestational SCH. Our results showed that the levels of TC, TG, LDL-C and TSH in patients with poorly controlled gestational SCH were abnormal and significantly higher than those in the SCH well controlled patients and patients without SCH. Pregnant women with hyperlipidemia are prone to atherosclerosis and cardiovascular disease due to abnormal blood lipids, which have a significant influence on pregnancy outcomes of the patients. Thyroid hormone replacement therapy is commonly used in clinical practice to improve maternal and fetal pregnancy outcomes of patients with SCH as thyroid hormone plays an important role in mediating lipid metabolism in the human body. The fetus can secrete thyroxine itself, but part of it still needs to be supplied by the mother. The decrease in maternal thyroxine will affect the nerves and bones of the fetus. Levothyroxine can reduce insulin resistance and avoid the occurrence of gestational diabetes mellitus, to avoid lipid metabolism disorders.

According to Tanguy f et al., the pathological mechanisms of SCH during pregnancy are associated with the resulting dyslipidemia and not only with the thyroid hormone levels.^14^ Zhou X et al.^15^ showed that vitamin D deficiency in the first trimester is associated with in an increased level of TSH in the first trimester, thereby aggravating subclinical hypothyroidism.^15^ The results of this study are consistent with this, but vitamin D is not observed this time, which is one of the limitations of this paper.

However, Batool S e*t al* showed that preoperational serum TSH levels are not associated with poor diagnosis in high stage of differentiated thyroid carcinoma, which may be related to the region of the study object.^16^ TC levels in serum are thought to be increased in patients with hypothyroidism and decreased in those with hyperthyroidism, reports have shown high levels of TC in pregnant women with SCH patients.^17^ TG are part of the body’s fats, and are maintained in a dynamic balance; however, during pregnancy, a high caloric diet and a reduced amount of exercise can cause excessive weight gain and increased lipid synthesis. In addition, stress during pregnancy alters the metabolism of pregnant women increasing their TG levels; the subnormal thyroid function of pregnant women with SCH further increases their TG levels.^18^ LDL-C is mainly synthesized in blood vessels, and high levels of the lipid are a risk factor for atherosclerosis. During pregnancy, the level of LDL-C can be increased due to diet and metabolic changes. In patients with SCH during pregnancy, the clearance and degradation rates of LDL-C are reduced due to the decreased level of thyroid hormone, resulting in an increase in the LDL-C level.^19^ It has been shown that the increases in serum TG and LDL-C levels are a direct impact of the hypothyroidism.^20^ Similar studies have also reported that elevated serum LDL-C levels may signal the presence of hypothyroidism in women.^21^ Cai Y et al.^17^ showed that the blood lipids levels and intestinal flora of pregnant women with hypothyroidism were significantly different from those of pregnant women without hypothyroidism, and that the same variables were associated with the pregnancy outcomes. Many studies proclaim serum TSH examination as the best index for the diagnosis of SCH, especially during the primary stages.^22^,^23^

Zhou J et al. showed that preeclampsia is more common in women with subclinical hypothyroidism, and that the TSH level of these patients is significantly higher than that of their healthy counterparts.^24^ Although high HDL-C levels in pregnant women can be caused by poor living habits (like an unhealthy diet and lack of exercise) during pregnancy, our results show only a small difference between the levels of pregnant women with SCH and those without it. Studies have shown that the probability of premature delivery caused by SCH during pregnancy is two times larger than that in pregnant women without it.^25^ Our study showed that the incidences of premature delivery, abortion and neonatal growth restriction in the SCH poorly controlled patients were significantly higher than those in the SCH poorly controlled patients and patients without SCH. Yang J et al. showed that the incidence of adverse pregnancy outcomes (early pregnancy abortion, premature delivery, pregnancy-induced hypertension, gestational diabetes mellitus, fetal growth restriction and low birth weight infants) in patients with SCH was significantly higher than that in pregnant women with normal thyroid function.^26^ Our results were basically consistent with Yang J et al.^26^ The occurrence of SCH during pregnancy can lead to abnormal weakening of thyroid hormone secretion, increase the risk of placental abruption, induce premature delivery, and also induce fetal organ growth restriction.

At the same time, after applying a Pearson analysis, we found that the levels of TC, TG and LDL-C were positively correlated with those of TSH and with the pregnancy outcomes; and, that the TSH levels were positively correlated with the pregnancy outcomes. Lin H et al.^27^ showed that TSH was significantly and positively associated with TG (P=0.03), which is consistent with this result, but they also found that serial TSH, free thyroxine (FT4) and free triiodothyroxine (FT3) in a Chinese population, and demonstrated that BMI ≥ 23kg/m2, material degree ≥ three and material age ≥ 30 years may increase the risk of thyroid dysfunction. Our study showed that there is no significant difference in age and gestational week between the two groups, which may be related to the selection of sample size.

Therefore, our study showed that SCH is associated with poor pregnancy outcomes, which indicating that full attention should be paid to pregnant women with SCH. Effective detection of blood lipids and TSH levels and timely correction of blood lipid disorders are of great significance for improving maternal and neonatal outcomes.

### Limitations:

First, our sample size was small, and the results have regional specificity due to factors such as dietary habits and geographical differences. Second, the retrospective nature of the study may weaken the validity of the findings. Third, the short-term and long-term effects of SCH on fetal cognition was not investigated in this study, which could be carried out in future research.

## CONCLUSION

The gestational levels of TC, TG, LDL-C and TSH in patients with poorly controlled SCH are increased. The levels of blood lipids and TSH are associated with the pregnancy outcomes. Ensuring the levels of blood lipids and TSH are within normal limits is expected to result in improved pregnancy outcomes.

### Authors’ contributions:

**JZ** and **HC** conceived and designed the study. **XD**, **WH** and **HZ** collected the data and performed the analysis. **JZ** and **HC** were involved in the writing of the manuscript and are responsible for the integrity of the study. All authors have read and approved the final manuscript.
